# A new point of view for mosaicplasty in the treatment of focal cartilage defects of knee joint: honeycomb pattern

**DOI:** 10.1186/s40064-016-2796-y

**Published:** 2016-07-26

**Authors:** Mehmet Fatih Erol, Ozgur Karakoyun

**Affiliations:** Department of Orthopedics and Traumatology, Namik Kemal University Medical School, Tekirdag, Turkey

**Keywords:** Mosaicplasty, Focal cartilage lesions, Knee, Honeycomb pattern

## Abstract

**Background:**

A focal full thickness cartilage lesion of knee joint is one of the commonly seen intraarticular pathologies among the joint cartilage problems. Osteochondral autograft transfer mosaicplasty is a method which has satisfactory outcomes among the treatment preferences of cartilage defects. In spite of the favorable clinical results there are some pitfalls of mosaicplasty treatment especially in cases of large defects. One of the major problems of the classical OAT mosaicplasty is the gap spaces between the plugs. The objective of the present study is to report the early clinical and radiological outcomes and to evaluate the clinical application of a new hexagonal osteochondral graft system (HOGS) with the hypothesis of improvement of the OAT mosaicplasty method with the use of hexagonally shaped plugs. The study has intended to answer two questions. (1) Does mosaicplasty with the new HOGS has favorable early clinical outcomes? (2) Is it possible to arrange the osteochondral autografts without leaving gap spaces by using hexagonal pattern in OAT mosaicplasty?

**Patients and methods:**

We designed a retrospective study to report analysis of early outcomes of the initial case series of OAT mosaicplasty by using a new HOGS. Six male individuals with the diagnosis of osteochondral defect and treated with HOGS and reached sixth month follow-up formed the basis of the study. The clinical status of the patients were evaluated with IKDC score. The radiological evaluations were carried out with direct X-rays and magnetic resonance imaging (MRI) studies. The mosaicplasty procedures were carried out via standard surgical method of classical OAT mosaicplasty by using the HOGS following a diagnostic arthroscopy in the same session. The inclusion criteria was a full thickness osteochondral lesion of femoral condyle between 1.5 and 6 cm^2^ and completion of 6 month follow-ups. The patients having deformities around the knee or major ligament lesions were excluded. MOCART scoring system was used for the evaluation of follow-up MRI findings. The clinical status of the patients were evaluated with IKDC scores.

**Results:**

The mean age of the patients was 40.8 (±5.2) years and the mean duration of the symptoms on presentation was 16 (±4.3) months. On the initial MRI studies mean defect area was measured 3.7 cm^2^ (±0.9) which was compatible with arthroscopic findings. During the surgical procedures it was possible to fill the defect area completely. The mean number of plugs in this series was 4.8 (±1.1). We did not faced with insufficient donor reserve problem in any of the cases. On the sixth month follow-up physical examination there was no limitation of the knee joint range of motion in the cases. All patients were able to bear weight on to their operated extremities without pain. The mean postoperative IKDC score improvement was 70.2 (±3.5) which shows significant improvement compared to preoperative scores. On the control MRI studies the bone integration of the hexagonal plugs were complete the cartilage continuity of the articular surfaces were intact and the grafts were well incorporated in all cases. The mean MOCART score on the 6th month MRI studies was 65.8 (±4.1).

**Conclusion:**

The early outcomes of OAT mosaicplasty with HOGS are comparable to studies on the classical mosaicplasty. According to our observations in this study we can say that the gap space left between the cylindrical plugs can be solved by using hexagonal prism shaped plugs.

## Background

A focal full thickness cartilage lesion of knee joint is one of the commonly seen intraarticular pathologies among the joint cartilage problems. The clinical picture can be ranged from knee pain, locking and swelling to severe functional impairment. Moreover, large defects are more likely to cause functional impairment which is especially important in young and physically active individuals and athlete (Bentley and Minas 2000). The treatment of focal cartilage lesion in the knee joint is still a challenging issue (Richter et al. 2015). Lesions >3 cm^2^ have worse results compared to smaller defects (Solheim et al. 2013). The management techniques of full thickness cartilage lesions can be listed as osteochondral autograft transfer (OAT) mosaicplasty, mosaicplasty with synthetic plugs whole tissue transplantation, autologous chondrocyte implantation, cell based scaffold treatment, tissue engineered collagen matrices with autologous chondrocytes and bone marrow stimulation via microfracture (Bedi et al. 2010; Clavé et al. 2016). Among them OAT mosaicplasty is a method which has satisfactory outcomes (Lynch et al. 2015; Hangody et al. 2010; Oztürk et al. 2006). This method is based on the transfer of osteochondral grafts harvested from the non-weight bearing areas of knee to the lesion area. Furthermore, in classical OAT mosaicplasty the autologous osteochondral grafts are cylindrical plugs of spongious bone covered with hyaline cartilage at one end. The advantages of OAT mosaicplasty are reconstruction of the articular cartilage with high percentages of hyaline cartilage, being a single stage procedure, low risk of disease transmission, and low cost. In spite of the favorable clinical results there are some pitfalls of mosaicplasty treatment especially in cases of large defects. Fibrocartilage filling of the gap spaces between cylindrical plugs and insufficient donor cartilage reserves are among the disadvantages of classical OAT mosaicplasty. The new hexagonal osteochondral graft system (HOGS) investigated in this study is designed with the inspiration taken from the honey bees. This hexagonal geometry is used in several fields of technology (Kalogirou 2004; Kaushika and Sumathy 2003).

The objective of the present study is to report the very early clinical and radiological outcomes and to evaluate the clinical application of a new HOGS with the hypothesis of improvement of the OAT mosaicplasty method with the use of hexagonally shaped plugs. The study has intended to answer two questions. (1) Does mosaicplasty with the new HOGS has favorable early clinical outcomes? (2) Is it possible to arrange the osteochondral autografts without leaving gap spaces by using hexagonal pattern in OAT mosaicplasty?

## Patients and methods

Before the study local ethical board approval has been obtained from Namik Kemal University Ethical Board (registration number: 2016.20.02.01). We designed a retrospective study to report analysis of early outcomes of the initial case series of OAT mosaicplasty by using HOGS. Twenty patients have admitted to our clinic with the diagnosis of the medial femoral condylar focal o and treated with OAT mosaicplasty by using HOGS in year 2015. Among them six male individuals who had reached sixth month follow-up formed the basis of the study. The clinical status of the patients were evaluated with IKDC subjective knee evaluation form which provides subjective evaluation of the patients experiencing knee related complaints. The scoring of the IKDC form was carried out by transforming the raw score to a 0–100 scale. The radiological evaluations were carried out with direct X-rays and magnetic resonance imaging (MRI) studies. The mosaicplasty procedures were carried out via standard surgical method of classical OAT mosaicplasty by using the HOGS following a diagnostic arthroscopy in the same session.

The inclusion criteria was a full thickness osteochondral lesion of femoral condyle between 1.5 and 6 cm^2^ and completion of 6 month follow-ups. The patients having deformities around the knee or major ligament lesions were excluded.

### Surgical method

**Fig. 1:**
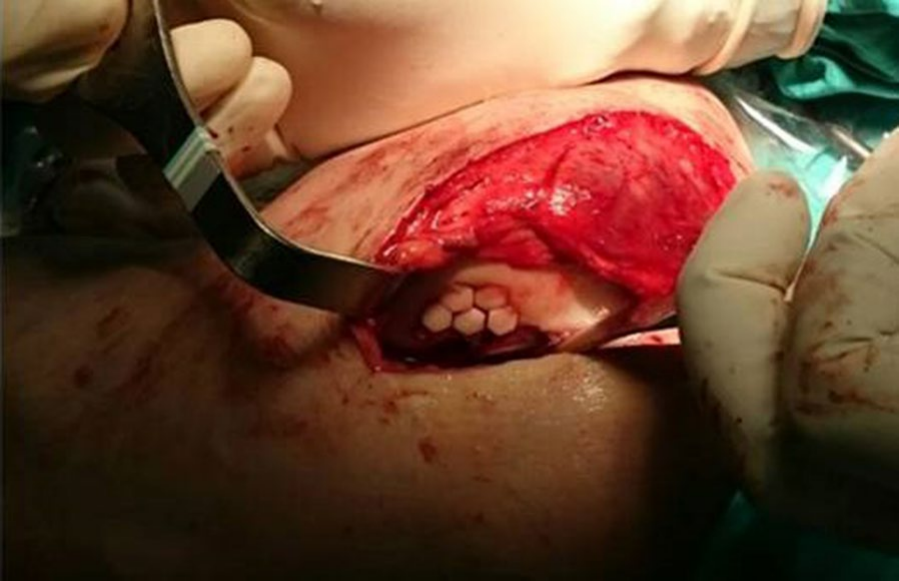
The view of the defect area at the end of the graft placement

All patients were undergone a diagnostic arthroscopy via standard anterolateral and anteromedial portals by using 30° scope following application of a pneumatic tourniquet onto the ipsilateral thigh region under anesthesia. During the arthroscopic examination the size of the lesion was recorded. Each case was also evaluated for any accompanying intraarticular knee pathology during arthroscopic examination. After that an anteromedial longitudinal incision was made. The osteochondral autologous hexagonal prism shaped plugs were harvested from the femoral site of non-weight bearing area of the ipsilateral patellofemoral joint with HOGS. The height of the prisms were 13 mm and the border length of the hexagons were 4 mm. The plugs were transferred to the host area one by one with the same surgical approach. During graft placement special care was taken for close side to side alignment of the plugs for the hexagonal arrangement without any gap (Fig. 1). At the end the stability was checked with full knee range of motion. In one patient with degenerative medial meniscus tear an arthroscopic partial meniscectomy was carried out before OAT mosaicplasty.

### Postoperative follow-up

All patients were discharged on the postoperative day one with active and passive knee range of motion and muscle strengthening exercises without weight bearing on the operated extremity. After that the patients were invited for outpatient controls weekly to follow-up the exercise compliance and functional status. At the postoperative fourth week full weight bearing was allowed. At the postoperative sixth week a control MRI study was carried out to evaluate the bony integration of the plugs and the cartilage continuity on the joint surface. MOCART scoring system was used for the evaluation of follow-up MRI findings. The clinical status of the patients were evaluated with IKDC scores.

## Results

The mean age of the patients was 40.8 (±5.2) years and the mean duration of the symptoms on presentation was 16 (±4.3) months. On the initial MRI studies mean defect area was measured 3.7 cm^2^ (±0.9) which was compatible with arthroscopic findings. One patient had an accompanying degenerative medial meniscus tear. Three of the patients had osteochondritis dissecans as the etiology and the other cases were idiopathic. None of the patients had a prior surgery related to the articular cartilage lesion.

The length of each harvested hexagonal prism shaped plugs was 13 mm and the length of the border of one hexagon was 4 mm. During the surgical procedures it was possible to fill the defect area of each case with the equally sized hexagonal autogenous osteochondral plugs which has 4 mm border length without leaving gap spaces. The mean number of plugs in this series was 4.8 (±1.1). We did not faced with insufficient donor reserve problem in any of the cases. All patients except one had well compliance to our exercise program. The patient needed to assistive physiotherapy on the second week of the operation. On the sixth month follow-up physical examination there was no limitation of the knee joint range of motion in the cases. All patients were able to bear weight on to their operated extremities without pain. Two patients had patellofemoral tenderness during the examination but it did not cause a limitation during daily activities. The mean postoperative IKDC score improvement was 70.2 (±3.5) which shows significant improvement compared to preoperative scores. On the control MRI studies the bone integration of the hexagonal plugs were complete the cartilage continuity of the articular surfaces were intact and the grafts were well incorporated in all cases. The mean MOCART score on the sixth month MRI studies was 65.8 (±4.1) (Table 1). Statistical Package for the Social Sciences (SPSS) for Windows (version 18.0).

**Table 1:** The list of the data and results of the patients

Patient	Age (years)	Mean duration of symptomes (month)	Etiology	Size of the defect (cm^2^)	Graft number	Accompanying lesion	IKDC improvement	6th month MOCART score
1	32	16	Osgteochondritis dissecans	4.4	7	None	78.6	60
2	43	19	Idiopathic	3.6	4	None	68.4	65
3	50	22	Idiopathic	2.6	3	None	64.7	70
4	35	13	Idiopathic	4.3	5	Yok	69.5	70
5	46	14	Osgteochondritis dissecans	5.2	6	Medail meniscus tear	74.5	60
6	39	10	Idiopathic	2.8	4	Yok	65.2	70

## Discussion

Full thickness cartilage lesions which can cause severe pain and functional impairment has no spontaneous healing potential. Therefore it is important to reconstruct such lesions especially in young people with high functional demands. OAT mosaicplasty is one of the modalities having higher grades of clinical evidence (Bedi et al. 2010). Hangody et al. published the preliminary report on OAT mosaicplasty and described the features of the method in 1997 (Hangody et al. 1997). Several studies revealed that OAT mosaicplasty has better outcomes compared to other techniques (Bentley et al. 2003; Robb et al. 2012; Krych et al. 2012). The early clinical and radiological results of the current series were comparable with the reports in the literature. Our very early results can be predictive for the long term results. However longer follow ups are necessary to give more definitive information.

Although classical OAT mosaicplasty has favorable clinical outcomes, gap spaces between the cylindrical plugs which end up with fibrocartilage filling remain as an issue to solve. When the coverage of a standard area with equally sized cylindrical plugs is calculated it can only cover 80 % of the defect. Radulescu et al. reported in their histological study the mean HC ratio of the treated area as 70 % (Rădulescu et al. 2008). In case of the use of differently sized plugs the gap spaces area can be reduced but cannot be eliminated (Hangody et al. 2004). There are a number of studies focused on the gap spaces. Ma et al. combined OAT mosaicplasty with bone marrow mesenchymal stem cell in their experimental animal study and concluded that this combination promotes integration of dead spaces between plugs (Ma et al. 2015). Chen concluded that tissue-engineered cartilage tissue can promote the integration of dead spaces between round plugs in their animal study (Chen 2014). The hexagonal design has intended to solve the gap interspace issue between the cylindrical plugs used in classical OAT mosaicplasty with changing the geometry of the autologous plugs. In the nature the honey bees produce their hive in combination of hexagonal cells. In 1999 Hales proved mathematically that hexagonal geometry is the way of build a construct with minimum amount of material without gaps (Hales 1999). Thus, the HOGS provides hexagonal arrangement with hexagonal prism shaped plugs which can be easily harvested and placed to the defect area with same surgical steps as in classical OAT mosaicplasty. Therefore we can presume that the learning curve of this technique would be short. Eventually, we observed in our study that application of OAT mosaicplasty with the new HOGS has no difference in surgical exposure and steps of the surgical procedure. However, the preparation step of the cartilage lesion has technically more difficult than harvesting the graft. Nevertheless, it was possible to align hexagonal prism shaped grafts without leaving interspaces in the defect area with the help of the geometrical nature of hexagons and obtain higher levels of coverage. We revealed according to these subjective observations HOGS can be an option to solve the gap space issue faced during the classical OAT mosaicplasty. But, it is obvious that future clinical or experimental studies have to prove the tissue quality of the cartilage tissue at the end of the treatment.

**Fig. 2:**
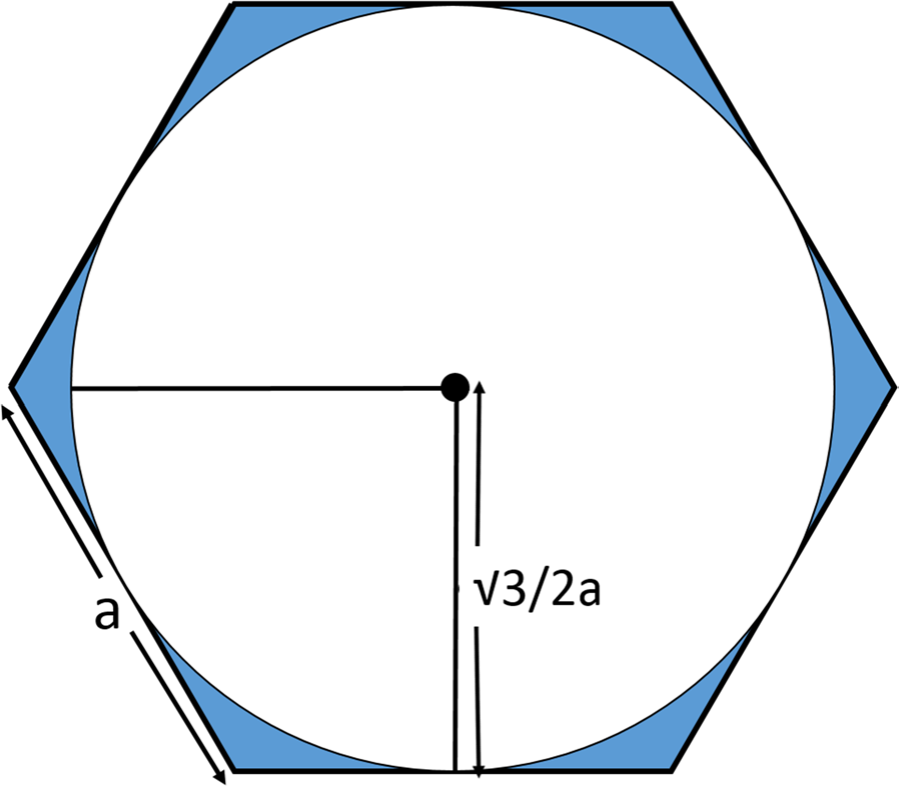
Drawing of a regular hexagon with a border with same length of a radius of a circle

Limited reserves of the autogenous osteochondral plugs are one of the disadvantage of OAT mosaicplasty. This creates an important problem particularly in large defects. Thaunat et al. studied on alternative donor areas in the knee joint in a study on cadavers (Thaunat and Beaufils 2010). They reported that posterior condyles of femur has comparable tissue quality to other donor sites but they did not recommend the routine use of posterior condyles as an alternative donor site because of technical difficulties. Furthermore, Shimada et al. selected the ribs as donor area and harvested costal osteochondral allografts for a large cartilage defect (Shimada et al. 2012). In this section the use of hexagonal plugs in OAT mosaicplasty can bring advantages. The hexagonal geometry of plugs harvested with HOGS can provide a higher amount of hyaline cartilage compared to circular plugs harvested from the same donor site. Mathematically, a regular hexagon with a border with same length of a radius of a circle has approximately 10 % higher surface area then the circle (Fig. 2). This calculation may reveal that harvesting hexagonal grafts rather than circular ones can increase the amount of harvested cartilage tissue. In our case series it was possible to harvest the hexagonal autologous plugs by using HOGS without any technical difficulty. Moreover, we did not encounter with autograft shortage in none of our cases. However this observations remain subjective and need to be proven with large series or experimental studies.

**Fig. 3:**
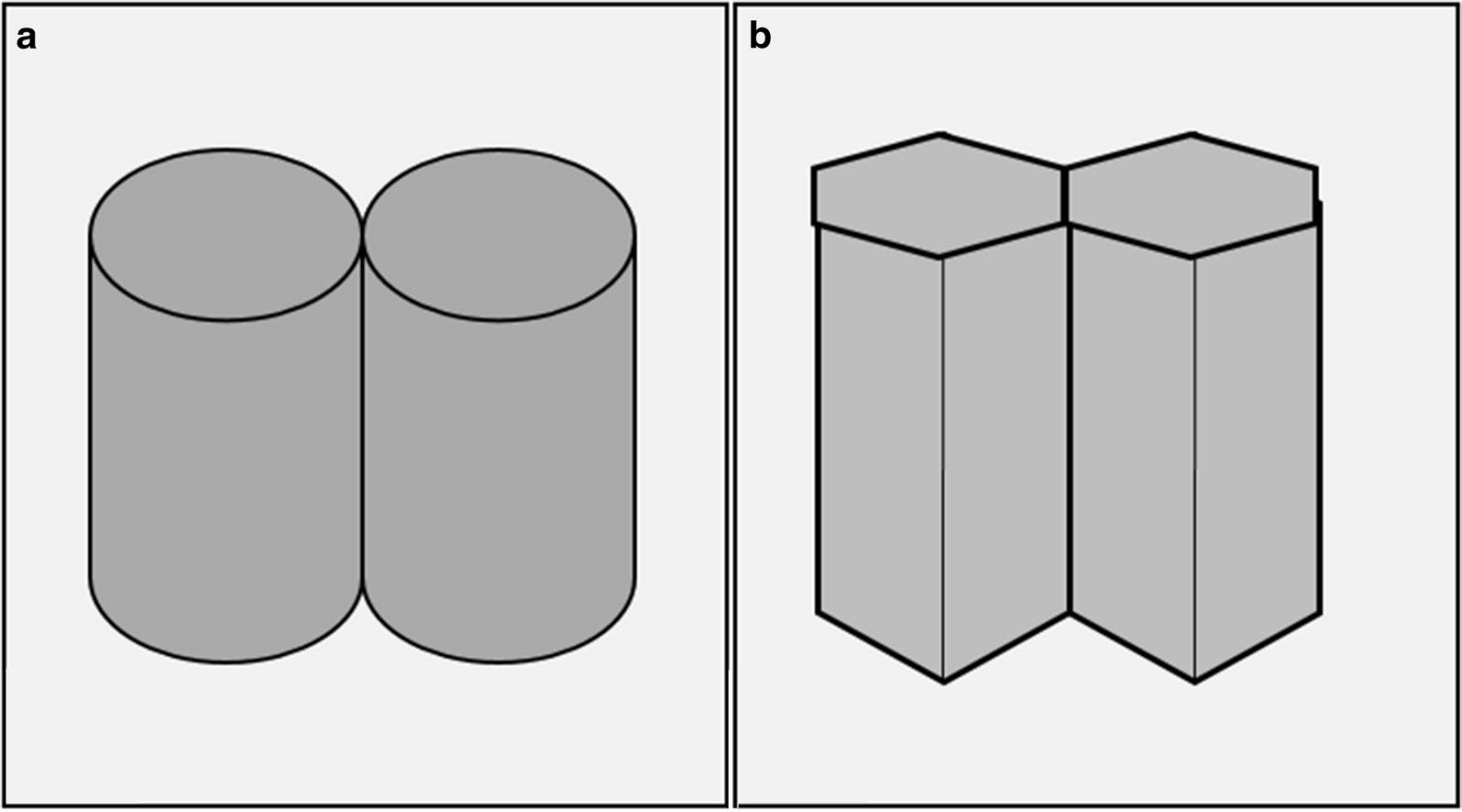
**a** The demonstration of the contact of the cylinders. **b** The demonstration of the contact of the hexagonal prisms

Additionally the osteochondral autografts should arrange with surface congruity and some stability. Sun et al. reported that the surface congruity effects the histological outcomes and incongruently aligned plugs caused instability of grafts and cartilage surface wear off in their experimental study on goats (Sun et al. 2013). Similarly, the stability and congruity of the osteochondral plugs on the host area is important for graft survival and maintenance of the cartilage healing process. In addition, special care and experience is necessary to achieve proper congruity and stability of the circular grafts on the chondral lesion during the surgical procedures. A cylindrical graft can have linear one dimensional contact with another cylinder (Fig. 3a). Therefore a long period is necessary before full weight-bearing on the operated extremity (Cognault et al. 2015). HOGS which we used in our study provided a two dimensional planar contact between the plugs (Fig. 3b). Consequently, there were no perioperative instability when during perioperative testing with full knee range of motion. Since the hexagonal plugs have linear contact, we were able to align the plugs much easier during our surgical procedures we allowed full weight bearing on the fourth week. On the sixth month follow-up MRI studies the bone integrity and surface continuities were intact, which was a clue for the sufficiency of the stability of the plugs. At the same time the follow-up MRI studies reveal that the treated joint surfaces were congruent.

It is clear that the present study has some limitations. A study on a larger patient population with longer follow-up would provide more reliable data on both clinical and radiological results. In this retrospective study no randomization was in place. A prospective randomized study containing control group would reveal more reliable results. The results of our series were promising. But for the effectiveness of HOGS on increasing HC coverage, histologic studies are obviously necessary. Either studies on second look arthroscopy or animal studies may provide useful information. The perioperative finding of stability that we observed remained subjective. This study involves calculations on the contact and stability of grafts. The biomechanical properties of hexagonal plugs have to be studied to obtain objective data. All surgical interventions in this study were carried out by one surgeon. To evaluate the technical applicability of the HOGS it is necessary to have multicenter studies including different populations as well as surgeons. Additionally the learning curve of this method can not be estimated with our subjective findings.

## Conclusion

The early outcomes of OAT mosaicplasty with HOGS are comparable to studies on the classical mosaicplasty. The system provides a different point of view and recommend solutions to gap space problem of classical OAT mosaicplasty. According to our observations in this study we can say that the gap space left between the cylindrical plugs can be solved by using hexagonal prism shaped plugs. But, larger clinical and experimental studies are necessary to have evidence based data on the effectiveness of the system.
